# 靶向GPRC5D治疗多发性骨髓瘤的研究进展

**DOI:** 10.3760/cma.j.cn121090-20240322-00109

**Published:** 2024-09

**Authors:** 嘉颖 安, 萌萌 潘, 皖雁 欧阳, 坚青 糜

**Affiliations:** 上海交通大学医学院附属瑞金医院血液科，上海血液学研究所，组学与疾病全国重点实验室，国家转化医学研究中心，上海 200025 National Research Center for Translational Medicine at Shanghai, Ruijin Hospital Affiliated to Shanghai Jiao Tong University School of Medicine, Shanghai Institute of Hematology, State Key Laboratory of Medical Genomics, Shanghai 200025, China

## Abstract

多发性骨髓瘤（MM）是一类由浆细胞克隆性异常增殖引起的恶性疾病，发病率占血液肿瘤的10％。近年来，随着靶向药物的研发和应用，MM的治疗取得了显著进展，但患者仍面临复发耐药的挑战。G蛋白偶联受体C类第5组成员D（GPRC5D）独立于B细胞成熟抗原（BCMA），在MM细胞中高表达，是继BCMA后极具潜力的新靶点。本文在重点介绍靶向GPRC5D治疗MM的疗效和安全性的同时，对靶向GPRC5D疗法的耐药复发机制及与BCMA双靶点序贯或联合治疗的时机也提出了展望。

## Abstract (en)

Multiple myeloma（MM）is a malignant disease caused by the abnormal clonal proliferation of plasma cells, accounting for 10％ of all hematologic cancers. In recent years, with the development and application of targeted drugs, a significant progress has been observed in the treatment methods of MM, but patients still face the challenges of relapse and drug resistance. Moreover, G protein-coupled receptor class C Group 5 member D（GPRC5D）is highly expressed in MM cells independently of B cell maturation antigen（BCMA）and is a highly promising target following BCMA. Aside from emphasizing the therapeutic efficacy and safety of targeting GPRC5D for the treatment of MM, this study also provides a prospective view on the mechanisms of drug resistance and relapse associated with GPRC5D-targeted therapies, as well as the timing of sequential or combined treatment strategies involving the dual targeting of both GPRC5D and BCMA.

多发性骨髓瘤（MM）是一种以骨髓中异常浆细胞克隆性增生为特征的恶性疾病，通常会引起贫血、骨痛、肾功能不全、高钙血症等临床症状。尽管蛋白酶体抑制剂（PI）、免疫调节药物（IMiD）和单克隆抗体等药物的联合应用改善了患者的生存及预后，但仍无法完全治愈MM，多药难治复发的患者预后很差。以B细胞成熟抗原（BCMA）为靶点的免疫疗法为该类患者带来了新的希望，其中嵌合抗原受体T（CAR-T）细胞产品西达基奥伦赛（Cilta-cel）在美国和中国复发/难治性MM（RRMM）患者中分别实现了97.9％和89.6％的有效率。然而，近半数患者在2年后仍面临复发，因此亟待寻求新的治疗靶点[1]–[2]。G蛋白偶联受体C类第5组成员D（GPRC5D）是目前继BCMA后极具潜力的新靶点，在RRMM患者中表现出令人惊喜的疗效，即使是BCMA CAR-T细胞治疗后复发的患者也能获得极高的反应率。本文主要总结了靶向GPRC5D治疗MM的疗效和安全性，对靶向GPRC5D疗法的耐药复发机制及与BCMA双靶点序贯或联合治疗的时机提出了展望。

GPRC5D是一种位于人类染色体12p13上的7次跨膜G蛋白偶联受体，在MM细胞上特异性高表达，其高表达与不良预后紧密相关[3]。GPRC5D在正常组织中有限表达，仅在皮肤毛囊和硬角化组织中的指甲或舌丝状、掌跖皮肤的成纤维细胞及唾液腺和扁桃体中的常驻浆细胞中低表达。此外，在下橄榄核和睾丸（生精小管）中也表达[3]–[6]。GPRC5D的表达独立于BCMA，因而不受BCMA缺失及既往治疗的影响，对于BCMA CAR-T细胞治疗后复发的患者依然有效[7]–[9]。

靶向GPRC5D的临床试验主要涉及双特异性抗体（BsAb）和CAR-T细胞疗法（表1、2）。目前，诱导多能干细胞衍生的嵌合抗原受体修饰的自然杀伤细胞（CAR-iNK）、抗体药物偶联物（ADC）及三特异性抗体（TsAb）等新型策略仍在临床开发阶段。

**表1:** 靶向GPRC5D的BsAb和CAR-T细胞疗法临床试验的疗效

临床试验	临床试验注册号	患者既往治疗情况			ORR	CR	VGPR	mPFS
		mLOT	三联难治率	五联难治率				
Talquetamab（BsAb）								
MonumenTAL-1	NCT03399799	6	79%	30%	70%和64%	23%和23%	57%和52%	10.2和7.8个月
MonumenTAL1-2	NCT04634552	5	74%和69%	29%和23%	74%和73%	10%和12%	59%和57%	9.3和13.0个月
RedirecTT-1	NCT04586426	5	78%	63%	全剂量：84%；RP2R：92%	–	–	–
TRIMM-2	NCT04108195	5	58%	63%	78%	45%	66%	19.4个月
RG6234（BsAb）								
RG6234	NCT04557150	5（IV）；4（SC）	62%（IV）；72%（SC）	36%（IV）；42%（SC）	71%（IV）；64%（SC）	4%（IV）；16%（SC）	25%（IV）；27%（SC）	PFS期未达到
CAR-T细胞疗法								
MCARH109	NCT04555551	6	16	17	71%	6例	10例	7.8个月
CC-95266（BMS-986393）	NCT04674813	–	100%	34%	86%	–	–	–
OriCAR-017	NCT05016778	5.5	–	–	100%	10例	4例	预计9个月PFS率87.5%
YK-GPRC5D BB-002	ChiCTR2100048888	4	–	–	91%	10例	4例	–

**注** BsAb：双特异性抗体；CAR-T细胞：嵌合抗原受体T细胞；mLOT：中位治疗线数；ORR：总有效率；CR：完全缓解；VGPR：非常好的部分缓解；PFS：无进展生存；mPFS：中位无进展生存期；IV：静脉注射；SC：皮下注射；RP2R：联合用药的推荐2期方案；–：未报道

**表2:** 靶向GPRC5D的BsAb和CAR-T细胞疗法临床试验的不良反应

临床试验	CRS	ICANS	血液学AEs			其他常见TEAEs		
			中性粒细胞减少	血小板减少	贫血	皮肤AEs	指甲AEs	口腔AEs
Talquetamab（BsAb）	
MonumenTAL-1	77%和80%	10%和5%	67%、36%	37%、23%	60%、43%	67%、70%	57%、27%	味觉障碍：63%和57%
MonumenTAL1-2	79%和72%（≥3级：2%/1%）	11%和10%	34%、28%	27%、27%	45%、39%	56%、68%	52%、43%	味觉障碍：48%和46%
RedirecTT-1	81%（≥3级：3%）	1例，3级	76%	–	60%	–	–	–
TRIMM-2	1～2级：78%	3例，1～2级	38%（3～4级占26%）	–	52%	皮肤剥脱：45%	–	味觉障碍：75%；口干：55%
RG6234（BsAb）	
RG6234	IV：82%；SC：79%；大多为1级；≥3级：IV和SC均为2.0%	IV：5例，≥3级1例；SC：7例，≥3级2例	IV：24%；SC：18%	IV：31%；SC：26%	IV：33%；SC：49%	IV：78%；SC：86%	IV：24%；SC：28%	–
CAR-T细胞疗法	
MCARH109	1～2级14例；4级1例（最高剂量）	1例，4级	94%	65%	35%	皮疹3例1级	指甲脱落11例	味觉障碍或口干2例，1级
CC-95266（BMS-986393）	1～2级55例；≥3级3例；5级1例	8例，3级2例	69%	30%	31%	24%	16%	味觉障碍/吞咽困难：3%
OriCAR-017	1级9例；2级1例	未发现	100%	90%	70%	干燥1例，2级；瘙痒2例，1～2级	指甲脱落3例，1级	–
YK-GPRC5D BB-002	1～2级25例	2～3级2例	100%	45%	52%	手足脱屑1例，2级	9例，1级	–

**注** BsAb：双特异性抗体；CAR-T细胞：嵌合抗原受体T细胞；CRS：细胞因子释放综合征；ICANS：免疫效应细胞相关神经毒性综合征；AEs：不良事件；TEAEs：治疗相关不良事件；IV：静脉注射；SC：皮下注射；–：未报道

一、BsAb

（一）BsAb结构类型及研发进展

1. Talquetamab（塔奎妥单抗）：Talquetamab是具有1个GPRC5D和1个CD3结合位点的双特异性IgG4抗体，由脯氨酸、丙氨酸和丙氨酸骨架（旨在减少Fc受体的结合）组成[7],[10]。研究显示，其募集活化T细胞以靶向杀伤MM细胞的能力与GPRC5D的表达水平呈显著正相关[7]。目前，美国食品与药品管理局（FDA）已批准Talquetamab用于既往至少接受过四线治疗的RRMM成人患者；欧盟也已批准其用于既往至少接受过三线治疗且在最后一次治疗中出现进展的RRMM成人患者[11]。

2. RG6324：RG6324（forimtamig）是一种具有两个GPRC5D结合位点和一个CD3结合位点的BsAb。在Ⅰ期临床试验中，无论是静脉注射还是皮下注射，RG6324在RRMM患者中均具有高度活性。目前，针对优化其剂量的研究正在进行中[12]。

3. BR109：BR109是一种采用“scFv（抗hCD3ε抗体）-Fab（抗GPRC5D抗体80A8）-Fc（人IgG1）”结构的非对称型BsAb，具有L234A/L235A突变（LALA）和旋钮进孔突变（KiH）。其结构与天然抗体相似，免疫原性较低，半衰期较长，在细胞和动物实验中均表现出较好的稳定性及抗肿瘤活性[13]。

4. LBL-034：LBL-034是一种靶向GPRC5D和CD3的人源化IgG1亚型非对称型BsAb。目前，LBL-034已获FDA和国家药品监督管理局（NMPA）批准，正在开展一项单臂、开放标签、多中心、剂量递增的Ⅰ/Ⅱ期临床研究（NCT06049290）。

5. BsAb5003：BsAb5003是一种采用“scFv-Fab-Fc”结构的人源化非对称型BsAb。与Talquetamab不同，其靶向GPRC5D的N端区域，并在流式细胞术中显示出强烈的靶标结合信号。BsAb5003在MM患者来源的骨髓样本和小鼠模型中均具有很好的疗效，与较低浓度的PI联用还可增强对MM细胞系的毒性[14]。

6. QLS32015：由齐鲁制药研发的注射用QLS32015在动物模型中表现出显著的细胞杀伤活性、良好的药代动力学特性和低毒性。目前已获NMPA批准，其Ⅰ期临床试验计划入组100例MM患者。

（二）BsAb单药临床试验

1. Talquetamab：MonumenTAL-1的Ⅰ期研究共纳入232例患者（102例静脉注射，130例皮下注射），其中164例由于疾病进展被迫终止治疗。Ⅱ期推荐剂量下，皮下注射患者中30例接受每周405 µg/kg的剂量，44例接受每两周800 µg/kg的剂量，两组具有相似的疗效和安全性，总有效率（ORR）分别为70％和64％，且大多数不良反应均为1～2级。但每周405 µg/kg剂量组的中位随访时间和中位无进展生存（PFS）期较长，分别为11个月和10.2个月，而每两周800 µg/kg剂量组分别为4.2个月和7.8个月。此外，既往接受过BCMA BsAb或CAR-T细胞治疗的16例患者的ORR为50％。此研究的局限性为皮下注射两个剂量组的患者数量较少，无法与其他临床试验直接比较[10]。

MonumenTAL-1的Ⅱ期研究共纳入288例患者，143例接受0.4 mg/kg皮下注射每周1次，145例接受0.8 mg/kg皮下注射每2周1次，显示两组疗效相似、安全性可控，在T细胞活化、重新分布和细胞因子诱导等方面与Talquetamab一致。常见的不良反应为细胞因子释放综合征（CRS）、皮肤指甲相关不良反应及味觉障碍，多为1～2级[16]。

一项单独研究Talquetamab皮肤指甲毒性的单中心回顾性试验共纳入14例接受Talquetamab单药或联合治疗的患者，其中12例患者出现皮肤毒性，大多数为1～2级。最常见的皮肤不良反应是手足综合征（50％），伴弥漫性炎性掌跖病变，呈双侧分布和继发性皮肤脱屑；14％出现皮肤裂隙；21％有干燥症；79％出现指甲变化，以脱甲病和博氏线最为常见，其次是甲营养不良、甲分离和化脓性肉芽肿。常见的口腔不良反应包括口干（71％）、味觉障碍（50％）[17]，可能与GPRC5D在舌状丝中表达有关[18]。上述提及的不良反应，均可通过使用合理的药物得到有效缓解，不会对患者的健康造成长期或不可逆的影响。

2. RG6324：在治疗的缓解率及不良反应等方面，RG6234均与Talquetamab相似[12]。既往接受过BCMA靶向治疗的10例静脉注射和11例皮下注射患者的ORR分别为50％和55％。然而，RG6234在T细胞的活化与增殖、细胞因子的产生及浆细胞的杀伤效果方面更为优越，特别是在注射后72 h内，能够高效清除浆细胞。在验证协同给药疗效时发现，RG6234与达雷妥尤单抗（Dara）或利妥昔单抗两药联合，或与Dara和泊马度胺（Pom）三药联合均会显著增强疗效。与Pom联合应用时，由于Pom具有更强的免疫调节作用，可以显著增强T细胞活化和细胞因子释放[19]。

（三）BsAb联合其他药物临床试验

1. RedirecTT-1：RedirecTT-1是一项Ⅰb期临床试验，应用Teclistamab和Talquetamab共靶向BCMA和GPRC5D，目的是通过克服耐药机制（如抗原逃逸）改善患者生存。此研究共纳入63例患者，在Ⅱ期推荐剂量下，ORR为92％，其中伴髓外疾病患者的ORR为83％。联合用药的安全性与两种单药一致，均处于可控范围内。最常见的不良反应为CRS、中性粒细胞减少症及贫血[20]。

2. TRIMM-2：目前正在进行的Ⅰb期TRIMM-2研究中，Talquetamab与Dara两药联用未出现额外的不良反应，具有良好的疗效以及可耐受的安全性。此研究共纳入65例患者，ORR为78％，其中66％的患者达到非常好的部分缓解（VGPR）及以上疗效。35例既往接受过BCMA靶向治疗的患者也有较好的疗效（ORR为74％）、可耐受的安全性及良好的中位PFS期。最常见的不良反应为免疫球蛋白降低（IgG<500 mg/d发生率为85％），其次为CRS以及味觉障碍，其发生率分别为78％和75％，均为1～2级[21]。

3. Monumen TAL-2：MonumenTAL-2是一项Talquetamab与Pom联合治疗的Ⅰb期研究，两者联合可使RRMM或治疗线数（LOT）≥2的患者获得快速、深度缓解。其初步疗效显示，每周1次和每2周1次队列的ORR相当，分别为86.7％和83.3％；但每周1次队列的完全缓解（CR）率及6个月PFS率更高，分别为60％和93.3％，而每2周1次队列仅为44.4％和88.9％[22]。

4. Monumen TAL-3：Monumen TAL-3是一项正在进行的随机、开放、多中心Ⅲ期试验，旨在评估Dara、Pom、地塞米松与Talquetamab联用的有效性和安全性。LOT≥1的810例RRMM患者以1:1:1的比例随机分配，接受28 d的TDP、TD或DPd方案，每隔一周皮下注射0.8 mg/kg的Talquetamab，其结果尚未发表[23]。

此外，Talquetamab还可与其他疗法联合用于MM患者，包括Ⅰ期TRIMM-3联合治疗试验（NCT05338775）、Ⅰ期日本单药治疗试验（NCT04773522）、Ⅲ期MajesTEC-7试验（NCT05552222）和Ⅱ期GEM-TECAL试验（NCT05849610）。目前正在探索将Talquetamab与抗CD38抗体、免疫调节剂和新型E3连接酶调节剂（CELMoD）（NCT06163898）联用，以进一步增强T细胞活性[24]。然而，BsAb联合其他药物的治疗策略在提高疗效的同时也会增加感染的风险。与单药治疗相比，全级别感染率增加了19％[25]。

二、CAR-T细胞

（一）MCARH109

MCARH109是一种第二代GPRC5D CAR-T细胞，具有人B细胞衍生的GPRC5D scFv片段、4-1BB共刺激域和CD3ζ信号域，其第一阶段剂量递增研究共入组17例患者，患者在接受4个剂量水平（25×10^6^、50×10^6^、150×10^6^、450×10^6^）的MCARH109治疗后均有较好的疗效，ORR为71％。接受靶向BCMA治疗后复发的10例患者ORR为70％。随着剂量的增加，疗效提高，不良反应也增加。接受（25～150）×10^6^剂量患者的ORR为58％，均未出现3级以上CRS、小脑病变及任何级别的免疫效应细胞相关神经毒性综合征（ICANS）；接受450×10^6^剂量的患者ORR为100％，但1例患者出现4级CRS和ICANS，2例患者出现3级不明原因的小脑病变[5]。

（二）OriCAR-017

POLARI是一项单中心、单臂、Ⅰ期临床试验，首次使用一种人源化双靶点纳米抗体串联的CAR-T细胞-OriCAR-017靶向GPRC5D的两个表位。该临床试验仅纳入10例RRMM患者，在患者体内静脉注射剂量递增的OriCAR-017（1×10^6^/kg、3×10^6^/kg、6×10^6^/kg），ORR高达100％。BCMA CAR-T细胞治疗后复发的5例患者疗效也均达到VGPR及以上。尽管ORR高达100％，所有患者也均达到微小残留病（MRD）阴性，但复发仍不可避免，对两例复发患者的骨髓进行检测，1例患者为GPRC5D阳性复发，另1例为GPRC5D阴性复发。OriCAR-017具有良好的安全性，CRS均为1～2级，未发生ICANS[27]。

（三）YK-GPRC5D BB-002

徐州医学院附属医院徐开林、李振宇教授开展了一项单中心、单臂、Ⅱ期临床试验。共纳入33例RRMM患者，输注剂量为2×10^6^/kg的CAR-T细胞后，ORR为91％，96％的患者MRD阴性。9例既往接受过BCMA CAR-T细胞治疗的患者均获得部分缓解及以上疗效。该CAR-T细胞产品安全性良好，在皮肤不良反应中，指甲不良反应发生率与Talquetamab相似，其他皮肤和口腔不良反应的发生率似乎低于其他GPRC5D靶向药物。该研究的不足之处为患者的LOT为4，低于其他CAR-T细胞临床试验[28]。

（四）BMS-986393

BMS-986393（CC95266）是一项剂量递增的多中心开放Ⅰ期研究，应用百时美施贵宝（BMS）研发的自体CAR-T细胞。共入组70例患者，分别接受25×10^6^/kg、75×10^6^/kg、150×10^6^/kg、300×10^6^/kg、450×10^6^/kg的CAR-T细胞，均表现出剂量依赖的持久疗效和降低的可溶性BCMA水平。四个剂量组的ORR为86％，其中既往接受过BCMA靶向治疗的32例患者ORR为75％。不良反应中较为突出的是，91％的患者血液学不良反应为3～4级[29]。

三、CAR-iNK细胞

FT555是一种诱导多能干细胞（iPSC）来源的CAR-iNK细胞，均一地表达CAR-GPRC5D（>90％）、hnCD16（>90％）和IL15RF（>90％）。虽然在FT555细胞表面未检测到CD38表达，但可与Dara联用，共靶向GPRC5D和CD38。在全面植入肿瘤的MM异种移植体内模型（CDX）中，FT555表现出强大的杀伤动力学和单次剂量的肿瘤清除，控制了长达42 d的MM进展，与未处理对照组相比，延长了37 d寿命[30]。

FT555与其他产品相比具有六种独特优势：①敲除了NK细胞本身表达的CD38，可避免抗CD38抗体消耗自身NK细胞；②具有靶向CD38^+^细胞的高亲和力，可与单克隆抗体结合并最大限度地发挥抗体依赖的细胞毒性；③其CAR可与非裂解CD16（hn CD16）协同抗肿瘤；④有独特的IL-15/IL-15受体融合蛋白，可促进细胞因子非依赖性功能；⑤可大规模生产，并现成可用[31]。

四、ADC

AZD0305是全球首个在RRMM中进入Ⅰ/Ⅱ期临床试验（NCT05647512）的GPRC5D-ADC，由抗GPRC5D单克隆抗体、蛋白酶可降解连接子和细胞毒素载荷单甲基奥瑞他汀E（MMAE）组成。体外研究表明，AZD0305具有与MM细胞系剂量依赖性高亲和力和强大的细胞毒性，在CDX中呈剂量依赖性抑制，其导致的溶酶体裂解情况可通过pHrodo染料观察[32]。

五、TsAb

GPRC5D的TsAb通过同时结合GPRC5D、BCMA和CD3三个靶点，引导T细胞定向攻击表达GPRC5D和BCMA的肿瘤细胞，具有低亲和力、高靶向激活力，有助于克服肿瘤异质性。目前研发的TsAb主要有JNJ-79635322（美国强生公司产品）、IBI3003（中国信达生物公司产品）、SIM0500（中国先声药业公司产品）以及MBS314（中国天广实公司产品），在临床前模型中均表现出优于BsAb的抗肿瘤活性和耐受性。强生公司生产的JNJ-79635322采用scFv融合形式，其剂量递增Ⅰ期研究正在进行中（NCT05652335）。信达生物公司的IBI3003采用了罗氏引进的CrossFab BsAb技术，重链之间则引入Knob-into-hole技术，是全球首款进入Ⅰ/Ⅱ期临床试验的产品，目前尚无数据发布。先声药业公司SIM0500的新药临床试验申请（IND）已获FDA批准。未来，通过深入研究TsAb的作用机制、安全性、优化制备工艺和质量控制进行更大规模的临床试验有望治愈更多MM患者。

六、靶向GPRC5D的挑战

尽管靶向GPRC5D在RRMM患者中显示出良好的治疗效果，但仍有部分患者复发耐药，需要进一步研究来揭示内在机制。研究表明，GPRC5D BsAb的获得性耐药可由多种因素介导，主要包括疾病负荷、抗原逃逸、T细胞耗竭[24]，也与GPRC5D位点的远端表观遗传沉默，及启动子和增强子区域的染色质协同沉默有关[26]。其中若初始反应良好的部分患者发生结构性染色体改变，如单核苷酸突变、框内缺失和平衡易位[33]，可能会导致GPRC5D双等位基因丢失或突变[34]，进而引起抗原逃逸的复发。

双重靶向BCMA和GPRC5D的CAR-T细胞疗法具有巨大的探索潜力。有研究探索了以下三个方案：①平行单靶点CAR-T细胞序贯联合；②单载体双顺反子双靶点CAR-T细胞疗法；③双sc Fv“单茎”CAR-T细胞疗法。结果显示，当BCMA阴性时，方案①和②均呈现出优于方案③的疗效；当两个抗原均表达时，双顺反子双靶点CAR-T细胞显示出更好的疗效[15]。目前，GPRC5D和BCMA的单靶点CAR-T细胞Ⅰ期同时给药试验（NCT05431608）及双靶点OriC-321 CAR-T细胞的Ⅰ期开放性临床试验（NCT05325801）正在进行中。

GPRC5D与BCMA靶点的序贯或联合治疗时机及理论依据也有待进一步探究。考虑到CAR-T细胞治疗出现严重感染风险、靶点突变及T细胞耗竭的发生率均较BsAb治疗低，有研究认为应首先使用CAR-T细胞疗法，将GPRC5D BsAb作为BCMA CAR-T细胞治疗早期（6～12个月）复发的最佳巩固或维持策略[35]，但制定治疗方案时还需要考虑患者的身体状况、肿瘤类型、分期及免疫状态等多个因素。

七、总结与展望

在GPRC5D CAR-T细胞研究中，较为突出的是OriCAR-017，其ORR较高，且不良反应较少，推测可能是由于双表位纳米抗体的CAR结构具有更高的亲和性。与CAR-T细胞疗法相比，BsAb的不良反应如味觉障碍、皮肤毒性、指甲变化以及感染的发生率似乎更高，而ICANS发生率较低，可能与BsAb的连续给药方案、对靶抗原的持续暴露，以及靶点的生物分布差异有关。以上临床试验均存在中位随访时间短、样本例数少的缺点，缺乏持久反应性和长期生存率方面的数据。因而，迫切需要进行全球多中心临床试验。

综上，GPRC5D作为RRMM的新靶点，显现出了令人鼓舞的疗效，且安全性良好。通过将靶向GPRC5D的免疫疗法与其他抗MM新药联合及应用双靶点CAR-T细胞，有望提高反应率，克服单一靶点治疗后抗原逃逸介导的复发，从而提升长期疗效。因此，进一步评估此靶点的最佳应用组合及时机，可为RRMM患者提供安全有效的个性化治疗方案。
